# Obstructive pancreatolithiasis in a cat with triaditis and concurrent hypercalcaemia

**DOI:** 10.1177/2055116921998494

**Published:** 2021-03-18

**Authors:** Frederik Allan, Anne-Lorraine Peschard, Luca Schiavo, Will Bayton, Davide Corbetta, Katie E McCallum

**Affiliations:** Queen’s Veterinary School Hospital, Department of Veterinary Medicine, University of Cambridge, Cambridge, UK

**Keywords:** Pancreatolithiasis, internal medicine, triaditis, hypercalcaemia

## Abstract

**Case summary:**

A 7-year-old neutered female domestic longhair cat was presented for further investigation of suspected hepatobiliary disease. Increases in serum 1,2-o-dilauryl-rac-glycero-3-glutaric acid-(6'-methylresorufin) ester lipase and hepatobiliary enzymes, with concurrent hypoalbuminaemia, were documented on blood biochemistry. Abdominal ultrasonography findings were consistent with acute pancreatitis with multiple pancreatoliths visualised within the pancreatic duct. Treatment for suspected triaditis was initiated with a hydrolysed protein diet, amoxicillin–clavulanate, hepatoprotectants and buprenorphine. Fifty-three days later, the patient presented with hypercalcaemia and obstructive pancreatolithiasis, and was euthanased. Post-mortem examination revealed severe chronic active pancreatitis with moderate chronic lymphocytic, plasmacytic cholangiohepatitis and mild chronic lymphocytic–plasmacytic duodenal enteritis (triaditis). Multiple calcium carbonate pancreatoliths present within the pancreatic ducts had resulted in pancreatic duct obstruction.

**Relevance and novel information:**

Pancreatolithiasis is a very rare condition in cats, with only five reports to date. In human medicine, pancreatolithiasis is often a sequala to chronic pancreatitis, seen in up to 50–90% of patients. However, in cats the aetiology of pancreatolithiasis, and indeed chronic pancreatitis, is poorly understood. This report describes a case of obstructive pancreatolithiasis in a cat with histopathological confirmation of triaditis and is the first report of hypercalcaemia in a cat with obstructive pancreatolithiasis. This further adds to the evidence base that pancreatolithiasis may have a similar pathogenesis to humans and can develop secondarily to chronic pancreatitis in cats.

## Introduction

Pancreatolithiasis is well reported in the human literature;^1–3^ however, only five feline cases, and sporadic bovine cases, have been documented in the veterinary literature.^1–2,4–8^

Pancreatolithiasis can occur in up to 50–90% of humans as a sequela to chronic pancreatitis, regardless of the aetiology.^3,9,10^ In most cats, as in humans, the pancreatic duct joins the common bile duct before entering the duodenum at the Ampulla of Vater, behind the duodenal papilla.^11^ Pancreatolithic obstruction of the pancreatic duct can result in the development of ductal, and subsequent parenchymal hypertension,^3^ leading to pain as the predominant clinical sign. The most frequently identified causes of chronic pancreatitis in humans are idiopathic chronic pancreatitis and chronic pancreatitis secondary to chronic alcoholism.^3,12,13^ Pancreatolithiasis has been reproduced experimentally in dogs by surgically induced partial obstruction of the pancreatic duct;^14,15^ however, the aetiology of pancreatolith formation in cats is poorly understood.

We present a unique case of a cat showing pancreatic duct obstruction secondary to pancreatolithiasis with histopathological evidence of triaditis and concurrent hypercalcaemia.

## Case description

A 7-year-old neutered female domestic longhair cat was referred to a specialist centre as an emergency for investigation of suspected hepatobiliary disease. The cat initially presented to the referring veterinarian with a history of vomiting, diarrhoea and lethargy. Routine serum biochemistry performed at the referring practice identified elevations in alkaline phosphatase (ALP), alanine transaminase (ALT) and bilirubin and, owing to clinical deterioration despite treatment with broad-spectrum antibiotics, the patient was referred for further assessment. Physical examination was unremarkable but limited due to patient temperament. The cat weighed 4.1 kg with a body condition score (BCS) of 5/9.

Initial investigations included a complete blood count (CBC), biochemistry and abdominal ultrasonography. CBC documented a moderate neutrophilia with occasional band neutrophils and toxic change observed on blood smear (Table 1). Serum biochemistry documented elevations in bilirubin, 1,2-o-dilauryl-rac-glycero-3-glutaric acid-(6'-methylresorufin) ester (DGGR) lipase, aspartate transaminase (AST), ALT, creatine kinase and glucose (Table 2). Feline immunodeficiency virus/feline leukaemia virus SNAP test (IDEXX) was negative, and serology for *Toxoplasma gondii* was negative, with IgG indirect fluorescent antibody test (IFAT) <50 (reference <50) and IgM IFAT <25 (reference <25).

**Table 1:** Complete blood count values at initial presentation and second presentation

Measurement	Day 0	Day 53	Reference interval
WBCs (×10^9^/l)	30.82*	24.22*	5.5–19.5
Neutrophils (×10^9^/l)	23.35*	18.67*	2.5–12.5
Lymphocytes (×10^9^/l)	4.7	3.7	1.5–7
Monocytes (×10^9^/l)	1.28	1.36	0–1.5
Eosinophils (×10^9^/l)	1.42	0.51	0–1.5
Basophils (×10^9^/l)	0.07	0.03	0–0.5
HCT (%)	28.5	16.8*	26–45
MCV (fl)	41.6	42.5	39–55
MCHC (g/dl)	34.0	33.9	30–36
RDW (%)	18.0*	15.7*	11.6–14.8
Platelets (×10^9^/l)	245.0	337.0	200–800
PCV (%)	31	17*	26–45
PP (g/l)	63	82*	60–80
Reticulocytes (×10^9^/l)	73.3	11.1	0–60

* Abnormal value

WBCs = white blood cells; HCT = haematocrit; MCV = mean cell volume; MCHC = mean cell haemoglobin concentration; RDW = red cell distribution width; PCV = packed cell volume; PP = plasma protein

**Table 2:** Serum biochemistry values at initial presentation and second presentation

Measurement	Day 0	Day 53	Reference interval
Urea (mmol/l)	5.5	5.0*	5.4–10.7
Creatinine (µmol/l)	95	90	56–153
Glucose (mmol/l)	9.3*	6.4*	3.9–5.8
Albumin (g/l)	23*	22*	25–43
Globulin (g/l)	40	58	24–47
Calcium (mmol/l)	2.08	3.00*	2–2.7
Phosphate (mmol/l)	1.06	1.31	0.9–2.1
ALT (IU/l)	266*	56	17–62
AST (IU/l)	79*	53*	0–51
CK (IU/l)	1061*	2403*	33–168
GGT (IU/l)	5.8	<0.3	0–10
ALP (IU/l)	76	4	10–93
Total bilirubin (µmol/l)	20.2*	3.1	0–11
Cholesterol (mmol/l)	3.00	1.59*	1.7–4.9
Lipase (DGGR) (IU/l)	36*	18	0–19
Serum amyloid A (µg/ml)	0.2	121.8*	<0.5
TLI (feline) (ng/ml)	Not performed	67	35–130

* Abnormal value

ALT = alanine transaminase; AST = aspartate transaminase; CK = creatine kinase; GGT = gamma-glutamyl transferase; ALP = alkaline phosphatase; DGGR = 1,2-o-dilauryl-rac-glycero-3-glutaric acid-(6'-methylresorufin) ester; TLI = trypsin-like immunoreactivity

**Figure 1:**
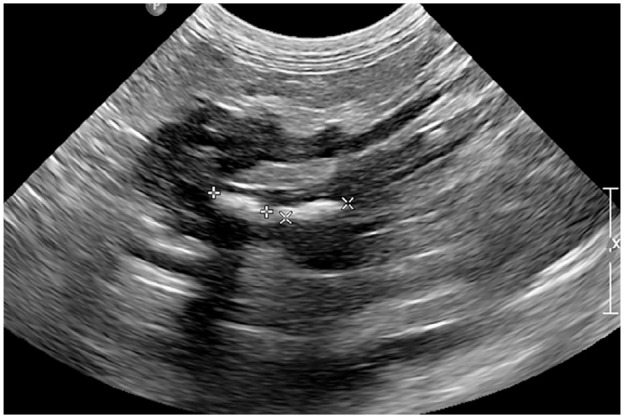
The pancreas is markedly hypoechoic and contains two large rounded hyperechoic shadowing structures (measured at 0.59 cm and 0.66 cm, respectively), identified as pancreatoliths

Abdominal ultrasonography revealed a mildly enlarged, hypoechoic pancreas surrounded by hyper-echoic mesentery. The pancreas contained multiple pancreatoliths (measuring between 0.4 cm and 0.7 cm) located both intraparenchymally and within the pancreatic duct (Figure 1). The wall of the pancreatic duct was markedly thickened at 0.68 cm (normal range 0.14–0.52 cm)^16^ and, at the point of joining the common bile duct, the pancreatic duct was dilated at 0.3 cm diameter (normal range 0.1–0.25 cm).^17^ The proximal common bile duct was dilated to 0.6 cm (normal diameter ⩽0.4 cm),^18^ with no visible structure obscuring the lumen, and the cystic duct was within normal limits. The duodenal papilla was thickened. The gallbladder was small and filled with anechoic fluid. The wall appeared thickened (0.20 cm; normal reference <0.1 cm),^19^ with an irregular mucosal surface. Underfilling of the gallbladder may have contributed to artefactual increase in wall thickness; however, the irregular luminal surface of the wall supported the suspicion for an underlying pathological process. The thickness of the wall of the jejunum was at the upper limit of normal at 0.27 cm (normal range 0.20–0.27 cm)^20,21^ with a hyperechoic mucosa. Mucosal hyperechogenicity in the small intestinal wall can be incidental (mild lymphoplasmacytic infiltrates have been found in the small intestinal wall of clinically normal cats)^22^ or may be observed with chronic enteropathies.^23^ The findings were consistent with acute pancreatitis with evidence of pancreatolithiasis, and were suspicious for concurrent cholecystitis or cholangitis, and enteritis. The patient was treated with a hydrolysed protein diet (Royal Canin Feline Hypoallergenic), a 4-week course of amoxicillin–clavulanate (12.2 mg/kg q12h PO [Clavaseptin; Vetoquinol]), a 4-week course of *S*-adenosylmethionine, vitamin E, silybin and vitamin K (50 mg q24h PO [Hepaticare; CEVA]) and buprenorphine (0.02 mg/kg q8h transmucosally [Vetergesic; CEVA]), with a recommendation for reassessment in 4 weeks. Antibiosis was continued owing to the presence of leukocytosis and toxic neutrophils,^24,25^ alongside a suspected risk of bacterial translocation.^26^

Fifty-three days after presentation, the cat was re-referred with an acute history of pyrexia, vomiting, tachypnoea and collapse. On presentation, the patient was tachypnoeic (respiration rate 80 breaths/min) and tachycardic (heart rate 200 beats/min) with a firm lobular mass palpable within the cranioventral abdomen. The patient had experienced significant weight loss since the last visit, weighing 3.75 kg with a BCS of 3/9. Rectal temperature was 40.5ºC. A thoracic-focused assessment for triage, trauma and tracking was performed and deemed unremarkable, and the patient was sedated for blood sampling, thoracic radiography, abdominal radiography and ultrasonography.

Complete blood count (Table 1) documented a moderate leukocytosis due to a neutrophilia consistent with systemic inflammation, and a moderate normocytic normochromic, non-regenerative anaemia, suspected to be associated with inflammatory disease. Serum biochemistry (Table 2) revealed markedly increased serum amyloid A (SAA) and creatine kinase, and decreased urea, albumin and cholesterol. There was a mild total hypercalcaemia; however, measurement of ionised calcium was not performed. Hepatobiliary enzyme activities, DGGR lipase, feline trypsin-like immunoreactivity (fTLI) and bilirubin were within the reference intervals.

**Figure 2:**
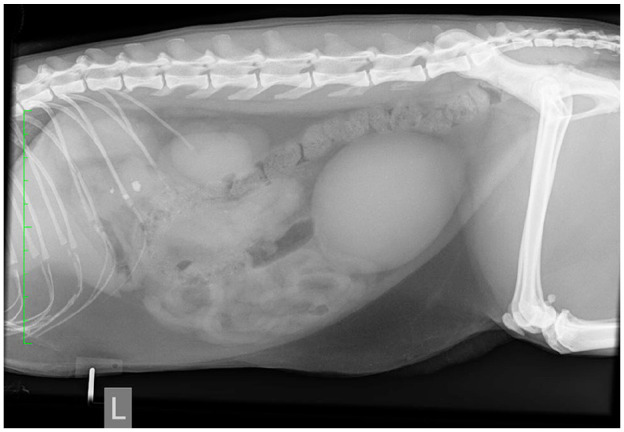
Left lateral abdominal radiograph. Three well-defined rounded mineral opacities are visible caudal to the stomach, in the region of the pancreas. Note the focal lack of serosal detail in the cranial abdomen, just caudal to the liver

**Figure 3:**
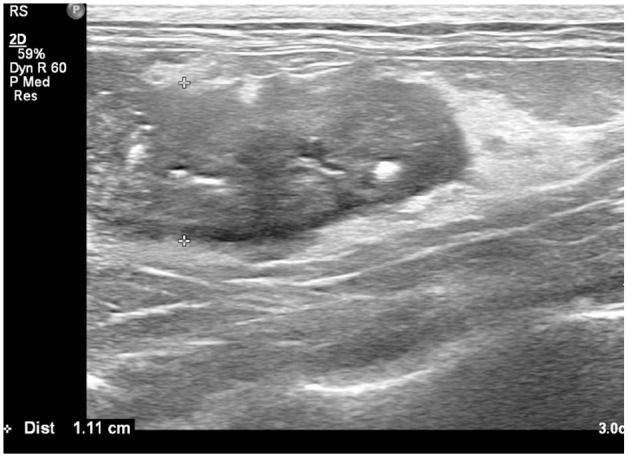
Left limb of pancreas at second presentation. Note the marked enlargement of the pancreatic limb, multiple hyperechoic structures within the pancreatic parenchyma and the markedly hyperechoic surrounding mesentery

**Figure 4:**
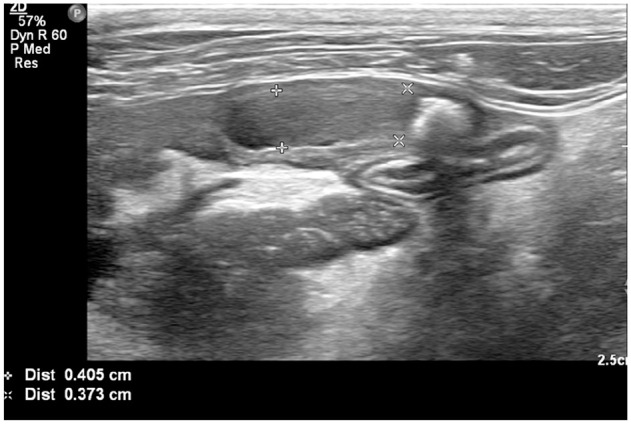
The measured tubular anechoic structure is a sagittal image of the pancreatic duct in the right limb of the pancreas. It is markedly dilated, filled with anechoic fluid and to the right of the image there is a large rounded hyperechoic shadowing structure within the pancreatic duct

Thoracic radiography documented an enlarged sternal lymph node. Abdominal radiography documented the presence of three large mineral opacities in the region of the pancreas (Figure 2). Abdominal ultrasonography documented a markedly enlarged, heterogeneous, hypoechoic pancreas with undulating margins and several pancreatoliths within the parenchyma (Figure 3), surrounded by markedly hyperechoic mesentery. A pancreatolith 0.4 cm in diameter was visible within the duct of the left pancreatic limb and a pancreatolith 0.5 cm in diameter was present within the duct of the right pancreatic limb, which was markedly dilated to 0.41 cm (Figure 4). A further pancreatolith 0.5 cm in diameter was present in the distal pancreatic duct at the level of the duodenal papilla, with marked dilation of the pancreatic duct proximal to the pancreatolith consistent with pancreatic duct obstruction. The common bile duct was within normal limits. A cholecystolith was present within the gallbladder. A small amount of anechoic free fluid was present surrounding the pancreas. Abdominocentesis cytology of the fluid revealed high numbers of non-degenerate neutrophils (82%), consistent with neutrophilic exudate, but no infectious agents were seen.

Owing to the evidence of pancreatic duct obstruction and the clinical status of this patient, conservative management was not considered to be a viable option. Surgical removal of the pancreatoliths with a partial or total pancreatectomy was discussed with the owner. However, based on compatible clinical and clinicopathological findings of tachypnoea, pyrexia, leukocytosis and markedly increased SAA, consistent with previously published criteria for clinical diagnosis of systemic inflammatory response syndrome,^27,28^ with potential recurrence due to incomplete pancreatolith removal, the client requested euthanasia and gave permission for a post-mortem examination to be performed.

**Figure 5:**
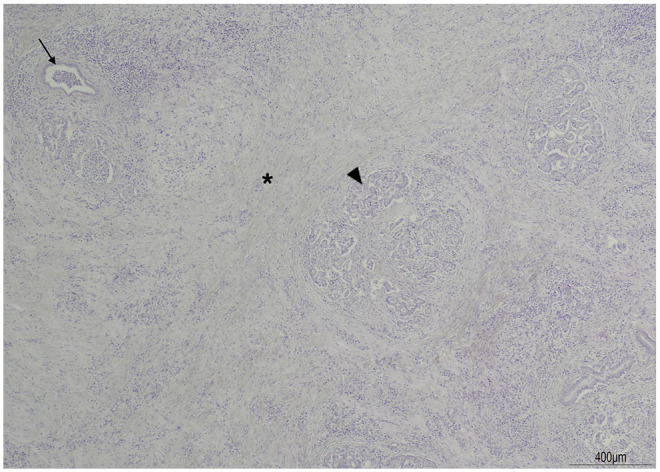
Pancreas. The pancreatic parenchyma is multifocally to coalescing replaced by abundant collagen (fibrosis) (asterisk), which is infiltrated and surrounded by moderate numbers of lymphocytes, plasma cells and viable, non-degenerate neutrophils, which expand the interlobular and intralobular septa. Pancreatic acini within the lobules are markedly reduced in size (atrophy) (arrowhead) and compressed by fibrosis and inflammatory cells. Few pancreatic acini are markedly ectatic (arrow) and contain moderate viable, non-degenerate neutrophils

**Figure 6:**
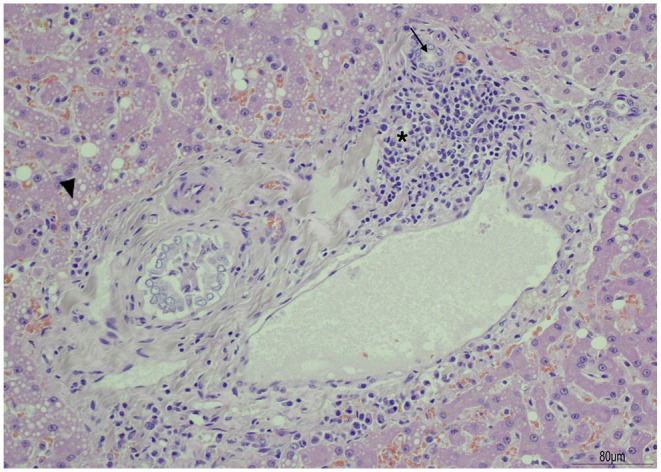
Liver. The picture shows a portal tract. The portal tract is expanded by moderate numbers of small mature lymphocytes and plasma cells (asterisk), which frequently surround and cross small bile ducts (arrow). The lumen of few bile ducts contains small amount of necrotic debris. Adjacent hepatocytes are mildly swollen and vacuolated (hepatocellular degeneration) (arrowhead)

The organs were harvested immediately after euthanasia and fixed in 10% neutral buffered formalin to prevent pancreatic autolysis. Post-mortem examination revealed macroscopic and histopathological changes consistent with severe chronic active pancreatitis with intraductal pancreatic calculi, pancreatic duct ectasia, necrosis and peripancreatic steatitis with fat necrosis. Marked pancreatic acinar atrophy affecting 95% of the pancreatic parenchyma was present and the inter- and intralobular septa of the pancreas were expanded by high numbers of small and medium-sized lymphocytes, plasma cells and neutrophils (Figure 5). No evidence of bacteria was present microscopically or via Gram staining. Moderate cholangitis was identified with evidence of biliary ductular proliferation and hyperplasia, portal fibrosis and a lymphoplasmacytic inflammation present in the biliary epithelium and lumen of the bile ducts and expanding the portal tracts (cholangiohepatitis) (Figure 6).^29^ Moderate chronic, lymphocytic–plasmacytic cholecystitis was evident with expansion of the lamina propria and submucosa by small lymphocytes and plasma cells. Mild chronic, lymphocytic–plasmacytic enteritis^30^ was also noted with multifocal reduction of the villi to approximately 75% of normal length, villar blunting, crypt distention, mucosal fibrosis, epithelial erosion and lymphoplasmacytic infiltrate expanding the lamina propria which, alongside the aforementioned cholangitis/cholangiohepatitis and pancreatitis, was consistent with triaditis. Mild interstitial, multifocal, lymphocytic–plasmacytic nephritis was observed with chronic multifocal interstitial fibrosis of the kidneys, consistent with non-azotaemic chronic kidney disease. The parathyroid and thyroid glands were unremarkable. Composition analysis of the pancreatoliths by infrared spectroscopy was consistent with 100% calcium carbonate composition (IDEXX).

## Discussion

Feline pancreatolithiasis is a rare disease, with only five previous reports in the literature.^1,4–7^ Ultrasonographic detection of pancreatoliths in a single cat was described within a case series; however, no further clinical aspects were described.^6^ In the remaining cases, pancreatolith composition was consistent with 100% calcium carbonate^1,5,7^ in three cats and a 50:50 mixture of calcium oxalate monohydrate and calcium phosphate^4^ in one cat. In human pancreatolithiasis, a reduction in pancreatic stone protein is thought to result in a supersaturation of calcium carbonate, which is then deposited over an inner nidus to form a pancreatolith.^3^ Given that most reported feline pancreatoliths to date are composed of 100% calcium carbonate,^1,5,7^ we postulate that a similar patho-mechanism may be involved in feline pancreatolith formation. Furthermore, this case adds weight to the association between pancreatolithiasis as a sequela to chronic pancreatitis, as reported in the human literature. Disease associations reported in cases of feline pancreatolithiasis include chronic pancreatitis, pancreatic nodular hyperplasia, pancreatic pseudobladder, duplicate gallbladder, inflammatory bowel disease and exocrine pancreatic insufficiency; the last may result from pancreatic duct obstruction.^1,4–7^

Previously documented haematological abnormalities in cats with pancreatolithiasis include non-regenerative anaemia and neutrophilia, although one case presented with mild neutropenia; serum biochemistry abnormalities included increases in AST and ALT activity, hypoalbuminaemia and hypocholesterolaemia.^1,4,5,7^ Interestingly, in 3/4 cases in which serum biochemistry values are reported, ALP, gamma-glutamyl transferase and bilirubin were within normal limits indicating that biochemical evidence of cholestasis is not always a significant feature in these cases. This hypothesis was supported in our case by the lack of extrahepatic biliary tract obstruction, wherein normal common bile duct dimensions were present ultrasonographically. Our findings agree with the existing literature, in which non-regenerative anaemia, neutrophilia, hypoalbuminaemia, hypocholesterolaemia and increased AST were apparent, and suggest that clinicopathological abnormalities are not specific to pancreatic obstruction due to pancreatolithiasis.Hypercalcaemia has not previously been reported in feline pancreatolithiasis. In fact, one previous case had hypocalcaemia, which was suspected to result from underlying pancreatitis^7^ due to sequestration of calcium in peripancreatic fat as a result of saponification, increased circulating free fatty acid concentrations, increased calcitonin concentrations secondary to hyperglucagonaemia and deficiency secondary to hypomagnesaemia.^11,24,31–33^ Unfortunately, ionised calcium was not measured in this case, representing a limitation of this report. Despite this, total hypercalcaemia with hypoalbuminaemia has been reported to be 98% specific in prediction of ionised hypercalcaemia in cats,^34^ which therefore suggests true hypercalcaemia in our case. It is possible that hypercalcaemia may have contributed to the progression of clinical signs associated with pancreatolithiasis due to an increase in stone quantity and/or size; however, it is important to be aware that pancreatolithiasis was present prior to the development of hypercalcaemia. Unfortunately, further investigations into the underlying cause of hypercalcaemia were not performed because the patient was euthanased. While idiopathic hypercalcaemia has been reported to be the most common aetiology for feline hypercalcaemia, followed by chronic kidney disease (CKD) and neoplasia,^35^ this is a diagnosis of exclusion. The lack of parathyroid hormone (PTH), PTH-related peptide (PTHrP) and vitamin D metabolite testing represents a limitation of this report. Despite the histological evidence of CKD, renal secondary hyperparathyroidism was deemed unlikely as this patient was non-azotaemic and normophosphataemic. Previous reports of feline pancreatolithiasis in which histopathology of the pancreas was performed were all consistent with chronic pancreatitis,^1,5,7^ which correlates with the histopathological findings in this case. However, this is the first report to describe histopathologically confirmed triaditis as a disease associated with feline pancreatolithiasis. Although triaditis was histopathologically confirmed post mortem, a limitation of this report is that cholecystocentesis for cytology and culture was not performed at initial presentation to confirm cholangitis, which can present without ultrasonographic abnormalities.^36^ Our findings and those of previous reports add to our driving hypothesis that chronic pancreatitis in cats may predispose to development of pancreatolithiasis, as is the case in human medicine.

While the underlying aetiology in the majority of cases of feline pancreatitis remains unknown,^24,25,32^ reported causes include pancreatic ductal obstruction, acute hypercalcaemia, pancreatic neoplasia, trauma, ischaemia and organophosphate intoxication.^32^ Associations with *T gondii* and certain viral diseases (such as coronavirus, parvovirus, herpesvirus and calicivirus infections) have been recognised but are uncommon.^11,24,37^ While we are assuming that chronic pancreatitis is the primary disease process leading to pancreatolithiasis, it is possible that acute hypercalcaemia and ductal obstruction due to the pancreatolithiasis itself led to acute exacerbation in this case.

Serum biochemistry derangements are often vague and non-specific for pancreatic disease; DGGR lipase has been reported to have excellent sensitivity (100%) and good specificity (63%) for acute pancreatitis but poor sensitivity (48%) in chronic pancreatitis.^11^ This could explain the normal lipase in our report at second presentation in the face of significant pancreatic pathology at post-mortem examination. A finding of low fTLI is suggestive of a >90% reduction of exocrine pancreatic function and is regarded the gold-standard test when diagnosing exocrine pancreatic insufficiency ^38,39^ but it is poorly sensitive for pancreatitis.^31,40^ The lack of decrease in fTLI, despite histopathological evidence of pancreatic acinar atrophy and ductal obstruction, is an interesting finding. It has been described that patients with EPI may have normal TLI if the pancreatic duct is obstructed;^41^ however, no cases have been reported in the literature. The mechanism of this is unclear; however, the authors postulate ductal obstruction may result in an inability of trypsinogen to pass into the duodenum and therefore it is subsequently reabsorbed into the circulation. Pancreatic inflammation secondary to obstruction could also play a role by causing increased leakage of trypsin and trypsinogen into the vascular space,^40^ resulting in subsequently increased amounts in the circulation. Interestingly, a study evaluating fTLI in cats with pancreatitis found 5/9 cats with pancreatic fibrosis to have fTLI within normal reference intervals, two of which had histological evidence of severe exocrine atrophy.^40^ The findings of normal fTLI in both our case and those in the aforementioned study highlight the possibility that fTLI can be unremarkable, despite histological evidence of pancreatic exocrine atrophy. Trypsinogen is typically cleared by glomerular filtration^40^ and another explanation for normal fTLI in our case is that our patient may have had reduced excretion due to CKD, leading to falsely elevated fTLI.^42^ Additionally, the histopathological report of 95% acinar atrophy is a subjective assessment based upon visualisation of the histological sections and may not completely reflect functional capacity of the pancreas.

Treatment options in human medicine for pancreatolithiasis consist of endoscopic management, which includes extracorporeal shockwave lithotripsy and stone extraction; surgical management, which includes pancreatic resection; and medical dissolution with trimethadione; however, the last is rarely used owing to limited evidence and significant side effects.^6^ Extracorporeal shockwave lithotripsy has been reported to achieve complete clearance in 80% of humans;^3^ however, this is not routinely available in veterinary medicine. Treatment options for pancreatolithiasis are limited in cats, likely as a consequence of both the paucity of available literature and rarity of this condition. Although surgical removal of pancreatoliths has been described in three cats, with survival times ranging from 9 days to 2 years postoperatively, with the third case lost to follow-up having survived 5 days to discharge,^1,4,7^ no other treatments have been reported.

## Conclusions

This report adds to the growing evidence base that pancreatolithiasis can occur in cats with chronic pancreatitis, either independent from or as part of triaditis, and is the first reported case of feline pancreatolithiasis with total hypercalcaemia and histopathologically confirmed triaditis. The relevance and aetiology of hypercalcaemia in this case is unknown; however, it is postulated that it may have contributed to progression of calcium carbonate pancreatolithiasis.
